# Adaptive leadership and safety citizenship behaviors in Pakistan: the roles of readiness to change, psychosocial safety climate, and proactive personality

**DOI:** 10.3389/fpubh.2023.1298428

**Published:** 2024-01-26

**Authors:** Hira Salah ud din Khan, Muhmmmad Salman Chughtai, Zhiqiang Ma, Mingxing Li, Di He

**Affiliations:** ^1^School of Management, Jiangsu University, Zhenjiang, China; ^2^Faculty of Management Sciences, International Islamic University, Islamabad, Islamabad, Pakistan; ^3^Managing People in Organizations, IESE Business School, University of Navarra, Barcelona, Spain; ^4^Research Center for Green Development and Environmental Governance, Jiangsu University, Zhenjiang, China

**Keywords:** adaptive leadership, readiness to change, psychosocial safety climate, proactive personality, safety citizenship behavior, healthcare, moderated mediation model

## Abstract

Challenging times have put organizations in a perilous and chaotic state that demands immediate resolution and calls for effective leadership to help navigate out of the crisis. In this context, we focused on psychosocial safety climate theory to investigate the influence of adaptive leadership on safety citizenship behaviors by looking at the mediating effect of readiness to change and the moderating impact of psychosocial safety climate and proactive personality, particularly in the Pakistani healthcare sector. To test the hypotheses, the data were collected from 397 employees working in the healthcare sector of Pakistan at two different times. The results of this study supported the model. The moderated path analysis revealed that psychosocial safety climate strengthens the direct effect of adaptive leadership on readiness to change, whereas the moderating impact of a proactive personality also strengthens the relationship between readiness to change and safety citizenship behaviors. Similarly, both moderators significantly moderated the indirect impact of adaptive leadership on safety citizenship behaviors via readiness to change. To conclude, the present study has significant implications for organizations and practitioners in both steady and uncertain environments.

## 1 Introduction

“Knowing how the environment is pulling your strings and playing you is critical to making responsive rather than reactive moves.” (Ronald Heifetz)

The world is currently faced with a chaotic situation that has sabotaged the normal infrastructure in terms of health, business, political environment, and ecology (1, 2). Human life has been critically affected by the inevitable changes due to unforeseen circumstances that call for restructuring or redesigning the organizational mold (3, 4). The whole system is collapsing, making it impossible to imagine how the new world will appear; this requires competent leadership to hold the system in place (5, 6).

Safety is of utmost value in healthcare organizations due to high-risk professions (7). Post-pandemic circumstances also increase the importance of safety behaviors as changes in professional working processes demand extraordinary safety behaviors to reduce the risk factors of any physical and psychological injury (8, 9). Safety citizenship behaviors (SCBs) have been described as voluntary behaviors aimed at enhancing employee group cooperative performance (10, 11). At this conjuncture, leaders’ proactive and prosocial behaviors ensure a safe working atmosphere (8), making it mandatory for organizations to bring adaptive leadership (AL) to demonstrate SCBs.

The involvement of individuals in the process of change is of utmost importance, and the concept of readiness to change (RTC) is widely recognized as a crucial factor in successfully implementing various change initiatives (12, 13). Administering change is not just an operational procedure but a vital competency for organizations that desire to stand tall in the future (14). Given this, RTC is a factor associated with leadership (15). Very few studies have investigated the impact of RTC, a pathway in leadership, and safety-related outcomes (16). These gaps are vital since AL aims to set the direction in turbulent situations (17), especially pandemics.

Psychosocial safety climate (PSC) indicates psychological circumstances at work because it is an expanded notion about a good and healthy workplace (18). The involvement and commitment of senior management show their guarantee to the employees through the activities of psychological health and safety policies and practices to prevent stress and uncertainty that may occur due to organizational change activities, which leads to higher level PSC at the workplace (19).

Employees’ safety behaviors can be triggered by several elements, and individual factors, i.e., proactive personality (PP), is one of them (20, 21). The PP trait is famous for taking the initiative at the workplace (20), and for higher SCB, individuals with these characteristics are essential. Highly proactive individuals find opportunities from problems and are not threatened by challenging and difficult circumstances (20), which usually occur during organizational change, especially in healthcare organizations.

**Figure 1:**
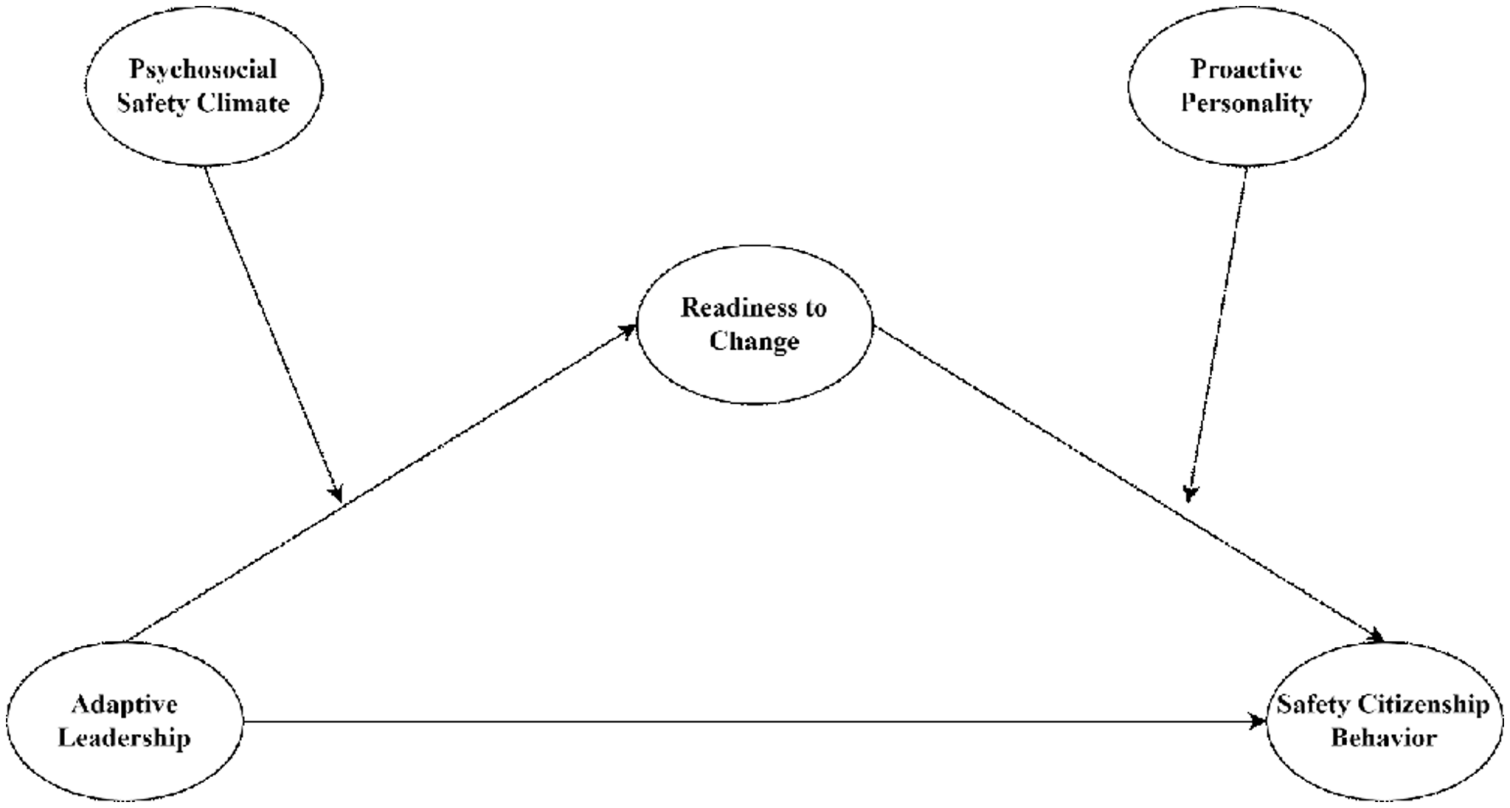
Research model.

Our study, which employed the PSC theory (22, 23), explains the impact of AL on SCBs and highlights the critical mediating role of RTC and moderating factors of PSC and PP that contribute to the leadership and organizational behavior literature in the following ways. Given the need for adaptive leaders to be helpful for the achievement of change objectives by giving new paths of working (24), we answer calls from Dartey-Baah et al. (25) and London (26) to explore the role of AL as a predictor of SCBs. Furthermore, this study also responds to the call from Sengupta et al. (27), which proposes the mediating impact of RTC that helps an organization to survive rather than struggle in the course of a global crisis by resulting in consistent SCB. Subsequently, the present study tests the moderating conditions of PSC leadership that have been overlooked to date, which Mansour (28) advocated in their recent review; our theorizing identifies an important PSC factor in employee behavior––that enhances the effects of AL on SCB. Next, by linking AL with SCBs and examining the moderating effect of PP, our study answers recent calls (29) to decipher mechanisms through which AL interactions impact SCBs in employees. Additionally, this study responds to the call from Tsandila-Kalakou et al. (30) by assessing the healthcare professionals’ adaptive capacity of employees and the role of contextual factors that could be used by hospitals to improve SCBs through educational efforts, individualized training, and motivational support. Our model presents the interplay of AL with PSC and PP through RTC for promoting SCBs in the healthcare sector, as illustrated in Figure 1.

## 2 Theory and hypotheses development

### 2.1 Psychosocial safety climate theory

This study’s pedestal was laid on the psychosocial safety climate (PSC) theory to support our hypotheses. This theory denotes “*organizational policies, practices, and procedures for protecting worker psychological health and safety*” (31). Scholars ordained that PSC determines the organizational structure policies and values that prompt communications and prevent work stress (32). Considering this, leaders are known to tune up the organizational climate through effective communication and stress management tactics (33). In this sense, AL is recognized as an effective leadership that consists of various strategies and conduct, which aids in a safe environment (34). A leader engages in facilitation activities to assist followers in learning, adapting, and changing their thinking, values, beliefs, and work routines after determining the sorts of difficulties (technical or adaptive) (35). The change of views and thinking by the leaders enables the subordinates to change their perceptions about organizational change and ultimately show their physical and psychological support (27, 36). Aligned with this, we posit that AL demonstrates helping behaviors and takes initiatives and safety measures that portray SCBs.

Furthermore, following the PSC theory, researchers conceded that a high PSC environment enables leaders to safeguard workforce well-being and ensure that the employees’ demands are manageable (37). Moreover, contemporary meta-analysis indicated that the health perils and motivational channels are mechanisms through which work demands and available resources are associated with a safe climate (38). Thus, we anticipated that a positive link between PSC and resources exists. Numerous studies have also endorsed PSC culture’s significance in organizations (22, 32, 39). Dollard and Bakker (31) established that high PSC was significantly linked to reduced work stress, emotional demands, and heightened skill discretion, which may be needed during the organizational change process. Ultimately, demands and resources conduit PSC’s influence level on perceived psychological health (31, 37). Moreover, Dollard and Bailey (40) asserted that PSC increases the coping abilities and personal energy of the individuals through which they can manage their job demands and reduce their stress, which may occur due to uncertainty of the organizational change. Thus, we examined the moderating role of PSC on AL and RTC.

Similarly, organizations with a substantial amount of PSC realize the need to make individuals feel protected to work in an atmosphere favorable to achieving organizational goals (18). Proactive individuals engage themselves by influencing the workplace environment to achieve higher performance (41, 42). Based on this notion, we argued that individuals belong to proactive personality traits; when they feel protected at the workplace, they participate more in extra-role behaviors (i.e., SCBs). In addition, the lens of PSC theory (22, 23) further explains that organizational climate encourages employees’ growth and development, and proactive individuals have the main characteristic for dealing with complex problems at the workplace with the aim of opportunities for them. It has been observed that PP plays a vital role in the demonstration of extra-role and safety behaviors (20, 43). Therefore, we expect that when individuals show their RTC with the support of PSC and AL, the employees of PP may bring more SCBs to the workplace, especially in healthcare organizations.

### 2.2 Adaptive leadership and safety citizenship behaviors

To deal with perplexing, uncertain, and shifting work environments, leaders must adapt (44) and possess the capacity to effectively communicate with followers (45) to bring about constructive change in vibrant work environments (46, 47). Shedding light on adaptive leaders’ roles exudes confidence and creates a favorable atmosphere where everyone feels secure (48). Researchers acceded that AL is an effective leadership approach in the field of management due to rapid organizational changes (49–51), such as in pandemics. Leaders respond to supportive gestures by executing citizenship behaviors and demeanor treasured by the organizations (44). Corroborating this, adaptive leaders exude confidence and create a favorable atmosphere where everyone feels secure (49). Organizations provide adequate resources to the managers to take appropriate action when threatening situations lead to safety measures (52). Studies of high-risk-oriented organizations demonstrate that AL is linked to effective environmental performance and safety (53). However, studies have investigated the phenomena related to safety and environmentally specific leadership, but AL and SCB associations require further exploration. Moreover, the PSC theory also advocates the importance of a healthy work environment that facilitates a safe climate and positive outcomes. Thus, we formulate the following hypotheses:

*H1:* Adaptive leadership is positively related to safety citizenship behaviors.

### 2.3 The mediating role of readiness to change

Leadership is a primary driver for organizational success, including functional and business performance outcomes (54, 55). A fundamental factor of successful organizational changes is that RTC relies primarily on the support of its leaders (56–58). Researchers have found that RTC has a favorable impact on organizational performance (24, 59). The concept of RTC means the degree to which workers consider how they are going to adapt to the change implementation in an organization (60). Workers constitute the primary stakeholders in an organization’s transformation and respond differently to the organization’s change implementation (61). Some staff members believe it will bring them happiness, pleasure, and benefits, while others may worry and feel they would suffer if the change is adopted in the organization (60). Furthermore, the literature indicates that RTC is critical for organizational change implementation and safety behaviors (62). Furthermore, the safety climate provided by the leaders encourages employees to engage in safety behaviors at work (63). As a result, AL has the ability to influence and encourage others to change their behavior correspondingly. Employees in healthcare organizations face different levels of stress and pressure, and workers must strive both physically and mentally to be safe and sound (38). These factors motivate adaptive leaders to shift employees’ views about change. The mindset of the leader redefines problem-solving by providing novel viewpoints on taking on roles, transforming obligations, and making sacrifices during difficult times (64). According to this, leadership could have an impact on RTC by influencing staff behaviors, which are unavoidable when preparing for change efforts, and subsequently influencing SCB. Thus, based on the above thrashing out, we hypothesize,

*H2:* Readiness to change mediates the relationship between adaptive leadership and safety citizenship behaviors.

### 2.4 Moderating role of psychosocial safety climate

Psychological safety climate is recognized as an essential ingredient of organizational climate that constitutes individuals’ perceptions about policies, procedures, and practices (31). Earlier studies elaborated that these perceptions are related to management support, effective communication, leader’s priorities, and participation regarding the workforce’s workplace safety and psychosocial health (22, 31, 32). According to Idris et al. (32) and Zadow et al. (65), a philosophical distinction exists between PSC and other notions, i.e., team psychological climate and safety climate. The focus of the safety climate is only on the policies, procedures, and practices regarding the physical safety of the employees at the workplace (66). In contrast, PSC focuses explicitly on the psychological health of the individuals at the workplace, covering a wide range of stressors that may occur due to interpersonal social relationships (32). Indeed, the management needs to emphasize PSC more than productivity (67). Furthermore, researchers expressed that PSC in an organization supports the change process, where leaders act according to the needs of the circumstances (22). Studies on organizational change have stressed leadership’s role concerning RTC (68). In line with this, leaders are considered agents of change that fulfill organizational needs (69, 70). It is also noted that high PSC aids psychological health and safeguards employees’ welfare (32). Moreover, PSC is a social factor determining workforce health and productivity (22). Accordingly, the PSC theory also supports the notion that a favorable work environment augments the leaders’ capability to become change agents (16). Because of these above arguments, we postulate the following hypotheses,

*H3:* Psychosocial safety climate moderates the relationship between adaptive leadership and readiness to change.

*H4:* Psychosocial safety climate moderates the indirect effect of adaptive leadership on safety citizenship behaviors via readiness to change.

### 2.5 Moderating role of proactive personality

The concept of PP pertains to an individual’s proactive efforts and actions aimed at successfully influencing their current circumstances, identifying potential opportunities, surmounting challenges and obstacles, and attaining their objectives while simultaneously exerting influence on their external events (41, 71, 72). Safety citizenship behavior is considered the employees’ discretionary safety activities to manage the risk at the workplace (11). Moreover, these safety activities are not required of employees in their job tasks, not for the formal reward or promotion, but they perform them voluntarily for smooth and effective functioning at the workplace (73). Different research findings show that PP can deal better with uncertain and adverse workplace situations, and by devoting their skills and abilities, they improve organizational well-being (74, 75). Moreover, proactive persons exhibit an optimistic orientation by independently initiating and doing preemptive initiatives to strategically plan and effectuate personal or environmental modifications in a favorable trajectory (71, 76). Numerous studies have reflected that individuals with higher levels of proactivity are more likely to achieve organizational and personal goals, i.e., innovative work behaviors (77), work engagement (78), professional identity (79), safety behaviors (20), proactive career behaviors (80), and cope with negative emotions, i.e., anger and stress (81). Based on this notion, we propose the moderating effect of PP between the relationship of RTC and SCBs, as we expect that proactive individuals during the organizational change process in the healthcare sector may demonstrate SCBs as they are willing cognitively to face challenges and want to learn from the environment. Thus, we hypothesize that:

*H5:* Proactive personality moderates the relationship between readiness to change and safety citizenship behaviors.

*H6:* Proactive personality moderates the indirect effect of adaptive leadership on safety citizenship behaviors via readiness to change.

## 3 Materials and methods

The present study is quantitative in nature, using a deductive approach to test the proposed hypotheses. For data collection purposes, a simple random sampling technique was applied, and close-ended questionnaires were distributed using the survey method to this study’s participants in two separate temporal time lags. The gap between the two time lags was 3 weeks to minimize the possibility of common method bias (CMB) (82). By following the recommendations by Podsakoff et al. (82, 83) and Rosenthal and Rosnow (84), we collected data from employees (i.e., subordinates and supervisors) to minimize the CMB and for the higher external validity of the data. For data collection purposes, healthcare sector institutions were randomly selected from the major cities of Pakistan (i.e., Rawalpindi, Islamabad, Lahore, Faisalabad, and Sargodha) in 2022 (February–April). Moreover, this study included data from different departments of healthcare institutions by collecting data randomly from employees who are directly linked with the health and care of the patients, i.e., operations, research, and development, and hospital services (front desks and pharmacy) (85, 86). At the same time, we excluded some departments, i.e., HR/admin, audit, budget, and planning. We randomly selected the employees of the healthcare sector of different cadres, i.e., medical trainees, medical officers, paramedical, and administrative staff. The reason behind the selection of these cadres is that these cadres are directly linked with patients’ health (in emergency and wards).

### 3.1 Procedures and participants

Respondents of this study participated in the survey voluntarily, and respondents (employees) were briefed about the research objectives and were assured of the information’s confidentiality. At the time lag one, we collected data from employees for PSC (first moderating variable) and RTC (intervening variable); in the second phase, we collected data from those employees who participated in the first phase to rate the perception of PP (second moderating variable) and AL (predicting variable) of their supervisors. We also collected data from immediate supervisors of employees who participated in both data collection phases to rate their subordinates’ SCBs (criterion variable).

At the time lag one, 350 survey questionnaires were distributed to subordinates in the first phase to collect their opinion about PSC (first moderating variable) and RTC (intervening variable), out of which 305 questionnaires were received and completed correctly. At time lag two, researchers distributed questionnaires to those subordinates who participated in the first phase for the collection of their opinions about PP (second moderating variable) and rated the characteristics of AL (predicting variable) about their respective supervisors/immediate officers, and at the end of the second phase of subordinates, 274 questionnaires were received, which were considered correct for further statistical analysis, so the response rate of subordinate questionnaires was 78.29%. Moreover, 200 questionnaires were distributed to supervisors/immediate officers to rate the SCBs (criterion variable) of their subordinates, and 123 questionnaires were received, which were completed from all aspects, so the response rate was 61.5%; the overall response rate was 72.18%. This study’s non-respondent rate was 27.82%, and we further performed a paired *t*-test using a 50/50 rule of early and late responses (87), and we cannot find any significant difference between both responses, which shows that there is no such influence of non-respondents on this study’s results. From the sensitivity analysis point of view, we perform single factor analysis suggested by Harman (88); according to Harman, if the cumulative % of total variance explained is less than 50%, then there is no issue of CMB, and the present study’s cumulative value was 28.35%. Moreover, we also performed inter-class correlation to check the comparability of the data between supervisor and subordinate responses, and we found a moderate degree of reliability where ICC was 0.610***, *p* < 0.001, with 95% confidence intervals (LL/UL-CIs = 0.533.677).

### 3.2 Measurement scales

The scales used in this study are validated in different Western and Eastern organizational contexts, but very few are tested in the Pakistani organizational contexts. For instance, the scales of adaptive leadership and psychosocial safety climate were tested in different Pakistani organizational contexts (24, 89–91). Moreover, the measurement of PP, RTC, and SCB are currently at an embryonic stage for assessment in the Pakistani healthcare sector. These scales are validated and widely used by numerous studies conducted in other cultures (20, 25, 59, 81, 92–96). Thus, this study is one of the very few to report these measures in the healthcare sector of Pakistan.

#### 3.2.1 Adaptive leadership

Adaptive leadership was assessed through a 15-item scale five items for each dimension, “Get on the Balcony,” “Identify the Adaptive Challenges,” “Regulate Distress,” adopted from Northouse (49). Sample items of the scale were “People recognize that my officer/manager has the confidence to tackle challenging problems” and “My officer/manager thrives on helping people find new ways of coping with organizational problems.”

#### 3.2.2 Readiness to change

Readiness to change was evaluated through a 6-item scale developed by Vakola (97). Sample items of the scale were “When changes occur in my organization, I believe that I am ready to cope with them” and “When changes occur in my organization, I always have the intention to support them.”

#### 3.2.3 Psychosocial safety climate

Psychosocial safety climate was assessed through a 12-item scale formulated by Hall et al. (67). Sample items of the scale were “Psychological well-being of staff is a priority for this organization” and “In my organization, the prevention of stress involves all levels of the organization.”

#### 3.2.4 Proactive personality

Proactive personality was assessed using a 6-item scale developed by Li et al. (98). Sample items of the scale were “I use opportunities quickly in order to attain my goals” and “Whenever something goes wrong, I search for a solution immediately.”

#### 3.2.5 Safety citizenship behaviors

Safety citizenship behaviors were measured using a 10-item scale established by Hofmann et al. (99) and Tucker et al. (52). Sample items of the scale were “He/she makes suggestions about how safety can be improved” and “He/she is assisting others to make sure they perform their work safely.”

#### 3.2.6 Control variables

This study includes some control constructs such as age, sex, education, and service. We controlled these confounding factors (demographics) during this study’s statistical analysis (correlation, direct, indirect, and moderation). We controlled these confounding factors by following the earlier studies (100, 101) and for the generalizability of the results.

## 4 Results

### 4.1 Demographic details

Table 1 demonstrates the demographic details of the participants (subordinates and supervisors).

**Table 1:** Demographic details (subordinates and supervisors).

Demographics		Subordinates		Supervisors	
Sex	Male	204	74.45	90	73.17
	Female	70	25.55	33	26.83
Age	20–30 years	85	31.02	51	41.46
	31–40 years	130	47.45	34	27.64
	41–50 years	45	16.42	21	17.07
	51–60 years	14	5.11	17	13.82
Education	PhD	7	2.55	4	3.25
	MS/M.Phil.	32	11.68	23	18.70
	Masters	114	41.61	39	31.71
	Graduation	121	44.16	57	46.34
Experience	1–5 years	63	22.99	17	13.82
	6–10 years	58	21.17	51	41.46
	11–15 years	86	31.39	39	31.71
	16–20 years	45	16.42	13	10.57
	More than 21 years	22	8.03	0	0.00

### 4.2 Confirmatory factor analysis, validity, and correlations

Before testing the proposed hypotheses of this study, we performed confirmatory factor analysis (CFA) and structural equation modeling (SEM) using statistical software, i.e., analysis of moment structures (AMOS). Table 2 shows the fit indicators of CFA, where *X*^2^/df is 2.05, and other fit values, i.e., GFI (0.88), AGFI (0.75), CFI (0.92), TLI (0.91), NFI (0.86), RMR (0.07), and RMESA (0.06); these all meet the generally accepted thresholds as suggested by Hu and Bentler (102), Tanaka (103), and Hair et al. (104). Moreover, in the SEM, the *X*^2^/df is 2.20, and fit indicators, i.e., GFI (0.86), AGFI (0.74), CFI (0.91), TLI (0.90), NFI (0.85), RMR (0.08) and RMESA (0.06) meet the thresholds as suggested by Hu and Bentler (102), Tanaka (103), and Hair et al. (104).

**Table 2:** Model measurement.

Measurement indicators	Acceptable range	CFA	SEM
CMIN/DF	1–3	2.05	2.20
GFI	>0.90	0.88	0.86
AGFI	>0.80	0.75	0.74
CFI	>0.90	0.92	0.91
TLI	>0.90	0.91	0.90
NFI	>0.90	0.86	0.85
RMR	<0.09	0.07	0.08
RMESA	<0.08	0.06	0.06

GFI, goodness of fit index; AGFI, adjusted goodness of fit index; CFI, comparative fit index; TLI, Trucker Lewis index; NFI, normative fit index; RMR, root mean square residual; RMESA, room mean square error of approximation; CFA, confirmatory factor analysis; SEM, structural equation modeling.

Table 3 demonstrates the values of discriminant and convergent validity using statistical techniques of the heterotrait-monotrait ratio of correlations (HTMT) and Fornell and Larker criterion. According to Hair et al. (105), composite reliability (CR) and average variance extracted (AVE) tests were performed to check the discriminant validity of the constructs, and for good validity, the values of CR and AVE must be 0.700 and 0.500. Table 3 shows the values of CR for all construct AL (0.986), RTC (0.874), PSC (0.923), PP (0.864), and SCB (0.919), which are in accordance with the threshold limits, whereas the values of AVE for all constructs AL (0.529), RTC (0.536), PSC (0.509), PP (0.515), and SCB (536) also meet the minimum threshold limit. The values of HTMT and Fornell and Larker criterion are also in line (as shown in Table 3, where diagonal values are higher than the others shown in rows and columns) with the recommendations of Hair et al. (105). Moreover, Table 3 also demonstrates the blindfolding values, which is a technique of recycling data for the cross-validation of all construct data, and if the values are zero or above zero, it shows the significance of predictive constructs (105).

**Table 3:** Validity.

Variables	Convergent validity		Blindfolding		
	CR	AVE	SSO	SSE	*Q*2
AL	0.986	0.829	4845.000	4845.000	0.000
RTC	0.874	0.536	1938.000	1841.489	0.050
PSC	0.923	0.509	3876.000	3876.000	0.000
PP	0.864	0.515	1938.000	1938.000	0.000
SCB	0.919	0.536	3230.000	2990.027	0.074

Variables	Heterotrait-monotrait ratio of correlations (HTMT)				
	AL	RTC	PSC	PP	SCB
**AL**					
RTC	0.322				
PSC	0.155	0.243			
PP	0.284	0.705	0.237		
SCB	0.120	0.332	0.225	0.416	

Variables	Fornell–Larker criterion				
	AL	RTC	PSC	PP	SCB
AL	0.911				
RTC	0.292	0.718			
PSC	0.142	0.213	0.714		
PP	0.265	0.582	0.215	0.732	
SCB	0.122	0.307	0.208	0.379	0.732

AL, adaptive leadership; RTC, readiness to change; PSC, psychosocial safety climate; PP, proactive personality; SCB, safety citizenship behaviors; CR, composite reliability; AVE, average variance extracted.

Table 4 shows the values of descriptive statistics, reliability, and correlations; the values of Cronbach alpha values are between 0.80 and 0.90, which shows good reliability of data and meets the threshold limit suggested by Sekaran and Bougie (106). Moreover, the correlation values of all study variables are positive and moderate as per the suggestions of Ratner (107). Table 4 shows the correlation values of all study variables where AL positively and significantly correlated with RTC (*r* = 0.255**, *p* < 0.01), PSC (*r* = 0.145**, *p* < 0.01), PP (*r* = 0.288**, *p* < 0.01), and SCB (*r* = 0.110*, *p* < 0.05). RTD positively and significantly lined with PSC (*r* = 0.203**, *p* < 0.01), PP (0.576**, *p* < 0.01) and SCB (*r* = 0.359**, *p* < 0.01). PSC positively and significantly associated with PP (*r* = 0.208**, *p* < 0.01) and SCB (*r* = 0.204**, *p* < 0.01), and PP is also positively and significantly correlated with SCB (*r* = 0.284**, *p* < 0.01).

**Table 4:** Model statistics and correlations.

Variables		Mean	SD	Alpha	1	2	3	4	5
1	AL	3.71	0.8547	0.98		0.255**	0.145**	0.288**	0.110*
2	RTC	3.67	0.7958	0.83			0.203**	0.576**	0.359**
3	PSC	3.17	0.8647	0.91				0.208**	0.204**
4	PP	3.71	0.7590	0.81					0.284**
5	SCB	3.63	0.7737	0.90					

AL, adaptive leadership; RTC, readiness to change; PSC, psychosocial safety climate; PP, proactive personality; SCB, safety citizenship behaviors, **p < 0.01, and *p < 0.05.

### 4.3 Hypotheses testing

To test the proposed hypotheses of this study (direct, indirect, moderation, and moderated mediation), we use 5,000 bootstrapping sample sizes through PROCESS-macro by following the suggestion of Hayes (108). Table 5 lists the results of direct, indirect, moderation, and moderated mediation. In the first phase, we analyze data to test out the direct effect hypothesis, which predicts that AL had a positive significant direct effect on SCB (*b* = 0.118, SE = 0.050, *t* = 3.357, *p* < 0.001, LL/UL-CIs = 0.081/0.117); therefore, the impact of AL on SCB is fully supported and thus prove *H1* of this study.

**Table 5:** Direct, indirect, moderation, and conditional indirect effects.

Relationships	Coeff	SE	*t*-value	*p*-value	LL/UL-CIs
**Direct effects**					
AL → SCB	0.118	0.050	3.357	0.001	0.081/0.117
AL → RTC	0.238	0.050	4.786	0.000	0.140/0.336
RTC → SCB	0.344	0.050	6.941	0.000	0.247/0.442
**Mediation effects**					
LOs → CSE → ACTC	0.082	0.021	3.848	0.000	0.047/0.131
**Moderation effects**					
AL → RTC	0.214	0.049	4.326	0.000	0.117/0.311
PSC → RTC	0.158	0.053	2.979	0.003	0.054/0.262
AL × PSC → RTC	0.131	0.062	3.499	0.001	0.090/0.151
RTC → SCB	0.299	0.069	4.316	0.000	0.163/0.436
PP → SCB	0.118	0.077	2.531	0.004	0.034/0.270
RTC x PP → SCB	0.174	0.066	3.129	0.001	0.055/0.204
**Conditional indirect effects (model-7)**					
AL → RTC → SCB conditional on PSC at +1 SD	0.087	0.025	2.722	0.007	0.029/0.156
AL → RTC → SCB conditional on PSC at mean	0.078	0.021	3.671	0.000	0.042/0.126
AL → RTC → SCB conditional on PSC at −1 SD	0.068	0.031	2.778	0.005	0.022/0.118
**Conditional indirect effects (model-14)**					
AL → RTC → SCB conditional on PP at +1 SD	0.086	0.030	2.876	0.004	0.038/0.156
AL → RTC → SCB conditional on PP at mean	0.074	0.023	3.171	0.002	0.036/0.127
AL → RTC → SCB conditional on PP at −1 SD	0.063	0.023	2.741	0.006	0.024/0.114

AL, adaptive leadership; RTC, readiness to change; PSC, psychosocial safety climate; PP, proactive personality; SCB, safety citizenship behaviors; UL/LL-CI, upper and lower-level class intervals.

In the second phase, we perform analysis to test our mediation hypothesis, and Table 5 illustrates the indirect effect results, which shows that RTC positively and significantly mediates the relationship with AL (*b* = 0.082, SE = 0.021, *t* = 3.848, *p* < 0.001, LL/UL-CIs = 0.047/0.131); these results support *H2* of this study because no zero was found been the values of upper/lower limit class intervals and explains a partial mediation of RTC. In other words, these results show that RTC is also a cause of the relationship between AL and SCB, which means AL increases the readiness level of individuals, which leads to the demonstration of SCB.

In the third phase, we performed statistical analysis to test both moderation hypotheses. Table 5 first shows the effect of predictor (AL), moderator (PSC), and interaction term (AL × PSC) on criterion variable (RTC), where a positive significant effect of interaction terms (AL × PSC) was found effect on RTC (*b* = 0.131, SE = 0.062, *t* = 3.499, *p* < 0.001, LL/UL-CIs = 0.090/0.151), which demonstrated that PSC strengthens the relationship of AL and RTC; thus, our *H3* was supported. In other words, these results show that when perceptions of individuals about AL and PSC were higher, it leads to RTC.

Table 5 further shows the moderation of second moderating variable (PP) with mediating variable (RTC) on criterion variable (SCB), a positive significant moderation (RTC × PP) effect found on SCB (*b* = 0.174, SE = 0.066, *t* = 3.129, *p* < 0.001, LL/UL-CIs = 0.055/0.204), which demonstrated that PP strengthens the relationship of RTC and SCB; thus, our *H5* was supported. In other words, these results show that when the readiness level of individuals was higher and they were at a higher level of their proactive personality, they demonstrated higher SCB.

**Figure 2:**
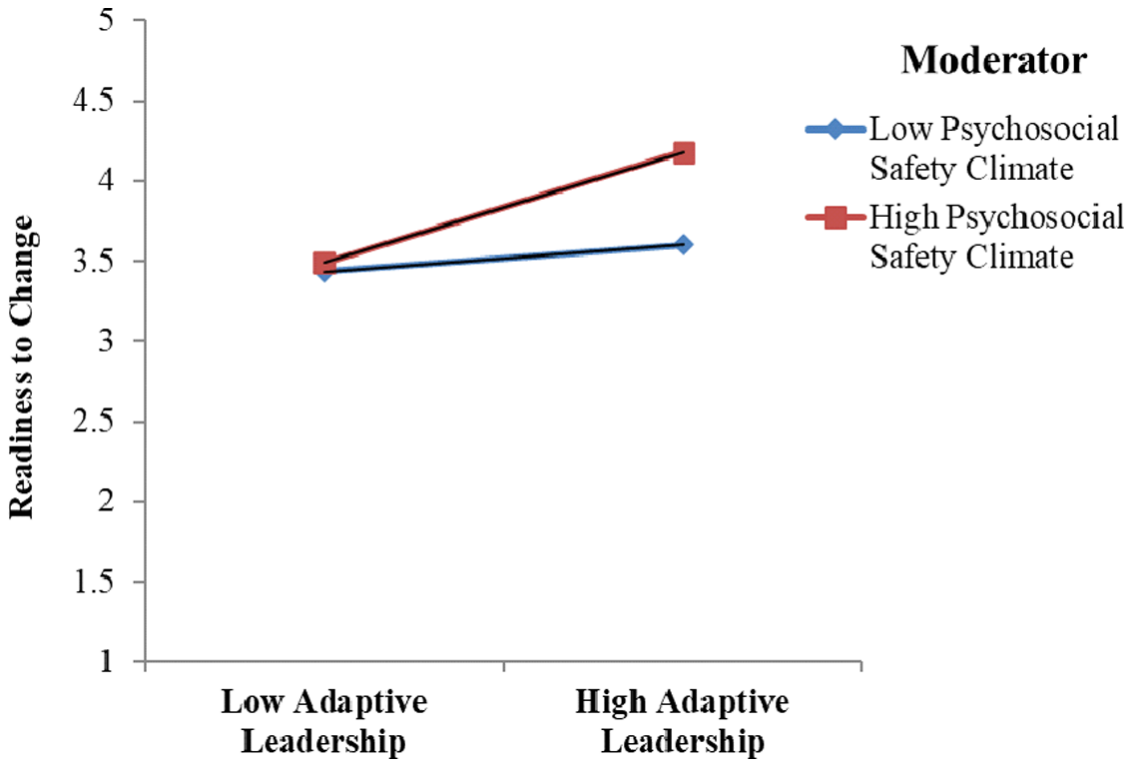
The interaction effects (AL × PSC) on RTC.

Furthermore, the interaction effects were illustrated by a graphical representation of RTC, as shown in Figure 2. The interaction term (AL × PSC) indicates that a higher level of PSC and AL enhanced the level of RTC.

**Figure 3:**
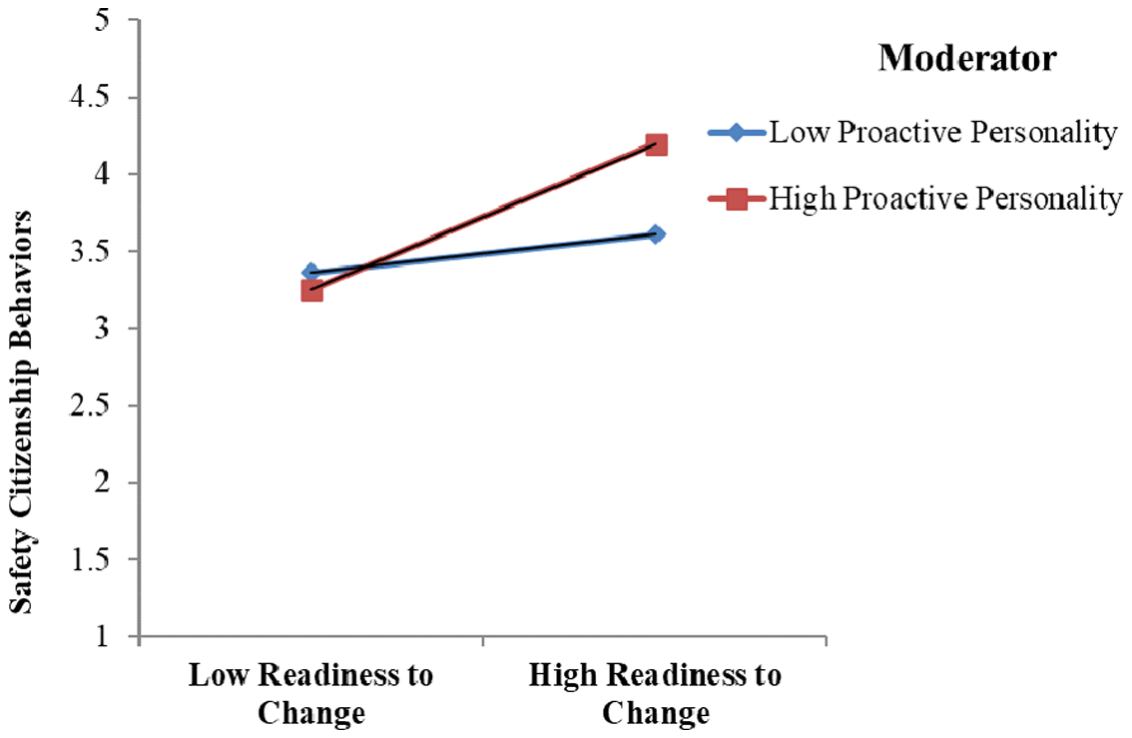
The interaction effects (RTC × PP) on SCB.

Furthermore, the interaction effects were shown by a graphical representation of SCBs, as shown in Figure 3. The interaction term (RTC × PP) illustrates that when individuals were at higher levels of RTC, their proactivity was also higher, and they demonstrated higher levels of SCBs.

Table 5 shows the values of conditional indirect effects (with PSC as moderator) which were calculated at ±1 SD. The values of conditional indirect effects were significant at +1SD (effect = 0.087, SE = 0.025, *t*-value = 2.722, *p* < 0.01, LL/UL-CIs = 0.029/0.156); these results also show that SCBs of individuals were higher via RTC when there was higher support of leadership, and they have higher perceptions about PSC because now zero was found between lower/upper class intervals. Thus, these results support our *H4*. In other words, higher support by the leadership (AL) and organizational environment (PSC) increases the readiness level of employees through which they become able to demonstrate higher SCB.

Moreover, Table 5 also shows the values of conditional indirect effects (with PP as moderator), which were also calculated at ±1 SD. The values of conditional indirect effects were significant at +1SD (effect = 0.086, SE = 0.030, *t*-value = 2.876, *p* < 0.01, LL/UL-CIs = 0.038/0.156); these results indicated that AL indirectly influences SCBs in the presence of a PP via RTC as no zero was found between the upper and lower limit confidence intervals. Thus, our *H6* was supported. In other words, higher support by the leadership (AL) increases the readiness level of employees, and personality characteristics (PP) enforce them to demonstrate higher SCBs.

## 5 Discussion

Using the theoretical lens of PSC theory (31), we hypothesize the research model of this study to test the impact of AL on SCBs in healthcare organizations. Moreover, we also investigate the mediating mechanism of RTC between AL and SCBs in turbulent times of healthcare organizations. Furthermore, we examine the moderating roles of PSC and PP between AL and RTC and between RTC and SCBs relationships, respectively. The first hypothesis of our study predicted that AL has a positive impact on SCBs, and the results of this study support the hypothesis. Moreover, these findings demonstrate that the role of leadership is imperative for the modification of subordinates’ attitudes and behaviors, especially in critical situations, i.e., organizational change (29, 89, 109). Through empathy, support, and motivation, such as AL, leaders can shape the SCBs of the employees (25, 44, 110). The second hypothesis of our study predicted the mediation mechanism of RTC between the relationship of AL and SCB, and this study’s findings demonstrated a partial mediation of RTC. These findings demonstrate that support of leadership (AL) using motivation increases the employees’ confidence, enabling them to face the uncertain circumstances of change (27, 111), and they demonstrate their willingness in the form of SCBs. The third and fourth hypotheses of this study predicted the moderating role of PSC, and the findings of this study provide support. The results explain that a higher level of PSC in the organization with the support of leadership (AL) directly increases the RTC and indirectly increases the SCBs. These findings are also in line with the earlier studies, which explain that a higher level of commitment and value by the management about the physical and psychological health of employees decreases the threat of uncertainty and enables them to deal with complex problems at the workplace (28, 112, 113). The fifth and final hypothesis of this study predicted the moderated role of PP, and the findings of this study support these hypotheses. The findings of this study explain that individuals who demonstrate a higher level of willingness to accept and support change policies and belong to the higher trait of PP perform SCB. These findings are also inconsistent with the earlier studies, which explain that proactive individuals, by their nature, modify the workplace circumstances for the betterment of the organization and the achievement of personal goals through the conversion of challenges into opportunities (20, 94, 114).

## 6 Conclusion

Most of the studies in management and leadership have encompassed steady work environments; the present pandemic and former critical events indicated that the right kind of leadership is imperative for organizational stability and workforce safety. In this sense, we proffered the influence of AL in the presence of contextual factors such as PSC, PP, and RTC that assist in amplifying the SCBs in challenging times. This aspect calls for organizations to have a changed outlook regarding the leadership roles for maintaining and establishing a pro-environmental workforce.

This study constitutes a framework to address the effect of AL on SCBs with the mediating effect of RTC and moderating effects of PSC and PP in the target population. The present study has been carried out on the healthcare employees working in different hospitals in Pakistan. This study revealed the influential role of AL in managing change and employees’ behaviors. Although several studies have been conducted on different types of leadership styles, there are very few studies that focused on AL in implementing change and assisting employees to display safety citizenship behaviors. This offers a significant contribution to the leadership and employee safety citizenship literature.

Furthermore, in developing countries such as Pakistan, healthcare is going through a multifaceted crisis, and we need leaders who can effectively and efficiently handle these complexities. More precisely, we require adaptive leaders more than ever before—people who support innovation, change, and experimentation. Therefore, this study emphasized the relationship of AL on SCBs with certain mechanisms in the healthcare sector of Pakistan. Moreover, the roles of RTC, PP, and PSC are neglected in healthcare institutions of Pakistan, and most studies are conducted on different sectors (24, 90, 115). As a result, the findings of this study can serve as a baseline for future interventions. It demonstrates the feasibility of such a survey in a developing country. It also lays the foundation for future research on aspects such as instrument validity and reliability in Pakistan. We look forward to studies investigating and promoting safety culture in developing nations. Additionally, this study reveals the importance of these aspects during recruitment or placing a proactive workforce in jobs in which roles may be vague. For new hiring, department placements, and project team launches, it could be useful to determine the proactive nature of applicants and use the findings for placement or selection. Moreover, proactive staff members could be assigned extra roles or work-related duties, which will enable them to solve problems on their own. The organizations, by supporting their members’ change-directed behaviors, will aid in the advancement of innovation and organizational change. Additionally, healthcare policymakers could pay close attention to the leadership style within their organizations since the current survey’s empirical findings indicate that an appropriate leadership style, such as AL, is necessary to cultivate an environment in which employees are encouraged to display their SCBs at the workplace. Moreover, this study unveiled the importance of SCBs for any emergency readiness, which is timely needed, especially in the healthcare sector of Pakistan.

### 6.1 Theoretical implications

This investigation is a minute addition to the existing knowledge body and the PSC theory. We go beyond the earlier studies and test the impact of AL as an organizational source for demonstrating SCBs. This study also uses PSC as a moderator, which also works as an organizational source with AL, enabling employees to fulfill complex job tasks during organizational change. Adaptive leaders, through their affective communication, motivation, and encouragement, increase the willingness and confidence of employees, whereas higher level PSC also gives psychological support to the employees to deal with uncertain circumstances; therefore, they demonstrate higher RTC and SCBs. In addition, we use PP as a moderator, a personal source of motivation through which individuals can face complex job tasks and challenges at the workplace, and here, adaptive leaders indirectly provide support to proactive individuals who also increase their willingness. Our moderated mediation models of PSC and PP facilitated us to keenly study the role of AL in forming SCBs in disruptive situations through RTC.

### 6.2 Practical implications

This study has many practical implications for management, researchers, and practitioners, focusing on leadership and safety perspectives and redefining the existing organizational structure in every situation. This study recommends that researchers and practitioners pay attention to phenomenal AL that helps to boot up the SCBs in critical times, especially in the healthcare sector in post-pandemic circumstances globally. Considering the current and future challenges, we brought to light the AL style to suit the globalized changing environment, as these leaders have the mastery of controlling the work arena effectively. Similarly, RTC is an imperative component for implementing change policies, especially in healthcare organizations where AL, through the encouragement, motivation, and empathic behaviors with their subordinates, enables them to display SCBs. Furthermore, it is suggested that organizations must focus on higher-level PSC so that their workforce supports the organizational change policies during critical times, as healthcare employees generally face a stressful workplace environment. Finally, it is suggested that organizations also focus on personality traits and, for that purpose, at the time of recruitment, they must test the employees’ personality traits to recruit a proactive workforce who can deal with crisis times willingly.

### 6.3 Limitations and future research directions

Regardless of its greater contribution to empirical, theoretical, and practical studies, this research has some limitations that should be focused on in future studies. First, we controlled the confounding factors, i.e., sex, age, education, and service; it is suggested that future researchers could also consider other confounding factors, such as department, marital status, and organization size. Second, in the present study, we collected data from employees (supervisor-subordinate rated) and used temporal separation during the data collection for predictor and criterion constructs to minimize the common method bias. It is suggested that future researchers may also adopt other data collection strategies to minimize the bias/sensitivity factor of the data as suggested by Podsakoff et al. (82, 83), i.e., tailoring of scale items to minimize the ambiguity of the words, which also reduce the social desirability bias and balance between positive and negative items of the scale. Thus, future studies could also investigate the possible interactions between two samples and comparability features. The third limitation of our study is that we investigated only SCBs as an outcome of AL, which allows researchers to explore other related outcomes, such as organizational performance and psychological well-being. In addition, this study examined two moderation mechanisms, PSC and PP; future investigations could focus on other instruments to explore further. Finally, this model could be tested in other cultural settings to divulge diverse findings.

## Data availability statement

The raw data supporting the conclusions of this article will be made available by the authors, without undue reservation.

## Ethics statement

The studies involving human participants were reviewed and approved by the Ethics Committee at Faculty of Management Sciences, International Islamic University, Islamabad, Pakistan. The patients/participants provided their written informed consent to participate in this study. The studies were conducted in accordance with the local legislation and institutional requirements. The participants provided their written informed consent to participate in this study.

## Author contributions

HK: Conceptualization, Investigation, Project administration, Writing – original draft, Writing – review & editing. ZM: Conceptualization, Funding acquisition, Investigation, Project administration, Resources, Supervision, Writing – original draft, Writing – review & editing. ML: Funding acquisition, Project administration, Resources, Supervision, Validation, Writing – review & editing. DH: Project administration, Resources, Writing – review & editing. MC: Project administration, Writing – review & editing.

## References

[1] Alam MA. Leading in the shadows: understanding administrative leadership in the context of COVID-19 pandemic management in Bangladesh. Int J Public Leadersh. (2020) 17:95–107. doi: 10.1108/IJPL-06-2020-0050

[2] Chang L Mohsin M Iqbal W. Assessing the nexus between COVID-19 pandemic—driven economic crisis and economic policy: lesson learned and challenges. Environ Sci Pollut Res. (2023) 30:22145–58. doi: 10.1007/s11356-022-23650-0, PMID: 36282386 PMC9593987

[3] Mamun MA Griffiths MD. First COVID-19 suicide case in Bangladesh due to fear of COVID-19 and xenophobia: possible suicide prevention strategies. Asian J Psychiatr. (2020) 51:102073. doi: 10.1016/j.ajp.2020.102073, PMID: 32278889 PMC7139250

[4] Yoganandham G. Making decisions for the future in a globalized society with erratic circumstances, collective action and transformational paths to enhance human development: an assessment. J Hum Resour Sustain Stud. (2022) 10:853–74. doi: 10.4236/jhrss.2022.104050

[5] Dirani KM Abadi M Alizadeh A Barhate B Garza RC Gunasekara N. Leadership competencies and the essential role of human resource development in times of crisis: a response to COVID-19 pandemic. Hum Resour Dev Int. (2020) 23:380–94. doi: 10.1080/13678868.2020.1780078, PMID: 36464814

[6] Khan HS Zhiqiang M Siddiqui SH Khan MAS. Be aware not reactive: testing a mediated-moderation model of dark triad and perceived victimization via self-regulatory approach. Front Psychol. (2020) 11:2141. doi: 10.3389/fpsyg.2020.02141 33041884 PMC7522326

[7] Derdowski LA Mathisen GE. Psychosocial factors and safety in high-risk industries: a systematic literature review. Saf Sci. (2023) 157:105948. doi: 10.1016/j.ssci.2022.105948, PMID: 32554212

[8] Bazzoli A Curcuruto M. Safety leadership and safety voices: exploring the mediation role of proactive motivations. J Risk Res. (2021) 24:1368–87. doi: 10.1080/13669877.2020.1863846

[9] Curcuruto M Conchie SM Griffin MA. Safety citizenship behavior (SCB) in the workplace: a stable construct? Analysis of psychometric invariance across four European countries. Accid Anal Prev. (2019) 129:190–201. doi: 10.1016/j.aap.2019.05.023, PMID: 31163325

[10] Hofmann DA Morgeson FP. Safety-related behavior as a social exchange: the role of perceived organizational support and leader–member exchange. J Appl Psychol. (1999) 84:286–96. doi: 10.1037/0021-9010.84.2.286

[11] Didla S Mearns K Flin R. Safety citizenship behaviour: a proactive approach to risk management. J Risk Res. (2009) 12:475–83. doi: 10.1080/13669870903041433, PMID: 33799997

[12] Rusly F Sun PY-T Corner JL. The impact of change readiness on the knowledge sharing process for professional service firms. J Knowl Manag. (2014) 18:687–709. doi: 10.1108/JKM-01-2014-0007

[13] Rusly FH Corner JL Sun P. Positioning change readiness in knowledge management research. J Knowl Manag. (2012) 16:329–55. doi: 10.1108/13673271211218906, PMID: 35498526

[14] Hussain ST Lei S Akram T Haider MJ Hussain SH Ali M. Kurt Lewin’s change model: a critical review of the role of leadership and employee involvement in organizational change. J Innov Knowl. (2018) 3:123–7. doi: 10.1016/j.jik.2016.07.002

[15] Muafi Fachrunnisa O Siswanti Y El Qadri ZM Harjito DA. Empowering leadership and individual readiness to change: the role of people dimension and work method. J Knowl Econ. (2019) 10:1515–35. doi: 10.1007/s13132-019-00618-z

[16] Metwally D Ruiz-Palomino P Metwally M Gartzia L. How ethical leadership shapes employees’ readiness to change: the mediating role of an organizational culture of effectiveness. Front Psychol. (2019) 10:2493. doi: 10.3389/fpsyg.2019.02493, PMID: 31798489 PMC6874171

[17] Arthur-Mensah N Zimmerman J. Changing through turbulent times—why adaptive leadership matters. J Stud Leadersh. (2017) 1:1–13.

[18] Law R Dollard MF Tuckey MR Dormann C. Psychosocial safety climate as a lead indicator of workplace bullying and harassment, job resources, psychological health and employee engagement. Accid Anal Prev. (2011) 43:1782–93. doi: 10.1016/j.aap.2011.04.010, PMID: 21658506

[19] Siami S Gorji M Martin A. Psychosocial safety climate and psychological capital for positive customer behavioral intentions in service organizations. Am J Bus. (2023) 38:1–21. doi: 10.1108/AJB-01-2022-0018

[20] Mo J Cui L Wang R Cui X. Proactive personality and construction worker safety behavior: safety self-efficacy and team member exchange as mediators and safety-specific transformational leadership as moderators. Behav Sci. (2023) 13:1–18. doi: 10.3390/bs13040337 PMC10136060 37102851

[21] Xia N Tang Y Li D Pan A. Safety behavior among construction workers: influences of personality and leadership. J Constr Eng Manag. (2021) 147:04021019. doi: 10.1061/(ASCE)CO.1943-7862.0002023

[22] Dollard MF Dormann C Idris MA. Psychosocial safety climate: a new work stress theory and implications for method In: Dollard M Dormann C Awang Idris M, editors. Psychosocial safety climate. Cham: Springer (2019). 3–30.

[23] Afsharian A Zadow A Dollard MF Dormann C Ziaian T. Should psychosocial safety climate theory be extended to include climate strength? J Occup Health Psychol. (2018) 23:496–507. doi: 10.1037/ocp0000101, PMID: 28857596

[24] Naseer S Chughtai MS Syed F. Do high-performance work practices promote an individual’s readiness and commitment to change? The moderating role of adaptive leadership. J Organ Chang Manag. (2023) 36:899–916. doi: 10.1108/JOCM-02-2023-0036

[25] Dartey-Baah K Quartey SH Adotey A. Examining transformational and transactional leadership styles and safety citizenship behaviors in the power distribution sector: evidence from Ghana. Int J Energy Sect Manag. (2021) 15:173–94. doi: 10.1108/IJESM-07-2020-0008

[26] London M. Causes and consequences of adaptive leadership: a model of leaders’ rapid responses to unexpected events. Psychol Lead Leadersh. (2023) 26:22–43. doi: 10.1037/mgr0000136

[27] Sengupta S Bajaj B Singh A Sharma S Patel P Prikshat V. Innovative work behavior driving Indian startups go global—the role of authentic leadership and readiness for change. J Organ Chang Manag. (2023) 36:162–79. doi: 10.1108/JOCM-05-2022-0156

[28] Mansour S. Psychosocial safety climate (PSC) In: Brough P Gardiner E Daniels K, editors. Handbook on management and employment practices. Cham: Springer (2022). 459–79.

[29] González-Mendoza JA Riaño-Solano M Sánchez-Molina J. Adaptive leadership and its competencies for times of crisis. J Lang Linguist Stud. (2022) 18:1049–61.

[30] Tsandila-Kalakou F Wiig S Aase K. Factors contributing to healthcare professionals’ adaptive capacity with hospital standardization: a scoping review. BMC Health Serv Res. (2023) 23:1–13. doi: 10.1186/s12913-023-09698-9 37496014 PMC10369840

[31] Dollard MF Bakker AB. Psychosocial safety climate as a precursor to conducive work environments, psychological health problems, and employee engagement. J Occup Organ Psychol. (2010) 83:579–99. doi: 10.1348/096317909X470690

[32] Idris MA Dollard MF Coward J Dormann C. Psychosocial safety climate: conceptual distinctiveness and effect on job demands and worker psychological health. Saf Sci. (2012) 50:19–28. doi: 10.1016/j.ssci.2011.06.005

[33] Wisittigars B Siengthai S. Crisis leadership competencies: the facility management sector in Thailand. Facilities. (2019) 37:881–96. doi: 10.1108/F-10-2017-0100

[34] Doyle A. Adaptive challenges require adaptive leaders. Perform Improv. (2017) 56:18–26. doi: 10.1002/pfi.21735

[35] Cao W Li P van der Wal C Taris T. Leadership and workplace aggression: a meta-analysis. J Bus Ethics. (2022) 186:347–67. doi: 10.1007/s10551-022-05184-0

[36] Somadi N Salendu A. Mediating role of employee readiness to change in the relationship of change leadership with employees’ affective commitment to change. Budapest Int Res Critic Inst J Humanit Soc Sci. (2022) 5:30–8.

[37] Dollard MF McTernan W. Psychosocial safety climate: a multilevel theory of work stress in the health and community service sector. Epidemiol Psychiatr Sci. (2011) 20:287–93. doi: 10.1017/S2045796011000588, PMID: 22201204

[38] Nahrgang JD Morgeson FP Hofmann DA. Safety at work: a meta-analytic investigation of the link between job demands, job resources, burnout, engagement, and safety outcomes. J Appl Psychol. (2011) 96:71–94. doi: 10.1037/a0021484, PMID: 21171732

[39] Hall GB Dollard MF Winefield AH Dormann C Bakker AB. Psychosocial safety climate buffers effects of job demands on depression and positive organizational behaviors. Anxiety Stress Coping. (2013) 26:355–77. doi: 10.1080/10615806.2012.700477, PMID: 22793792

[40] Dollard MF Bailey T. Building psychosocial safety climate in turbulent times: the case of COVID-19. J Appl Psychol. (2021) 106:951–64. doi: 10.1037/apl0000939, PMID: 34383511

[41] Bateman TS Crant JM. The proactive component of organizational behavior: a measure and correlates. J Organ Behav. (1993) 14:103–18. doi: 10.1002/job.4030140202

[42] Baba VV Tourigny L Wang X Liu W. Proactive personality and work performance in China: the moderating effects of emotional exhaustion and perceived safety climate. Can J Adm Sci. (2009) 26:23–37. doi: 10.1002/cjas.90

[43] Lai F-Y Lin C-C Lu S-C Chen H-L. The role of team–member exchange in proactive personality and employees’ proactive behaviors: the moderating effect of transformational leadership. J Leadersh Organ Stud. (2021) 28:429–43. doi: 10.1177/15480518211034847

[44] Yukl G Mahsud R. Why flexible and adaptive leadership is essential. Consult Psychol J. (2010) 62:81–93. doi: 10.1037/a0019835

[45] DeRue DS. Adaptive leadership theory: leading and following as a complex adaptive process. Res Organ Behav. (2011) 31:125–50. doi: 10.1016/j.riob.2011.09.007, PMID: 24589246

[46] Uhl-Bien M Arena M. Leadership for organizational adaptability: a theoretical synthesis and integrative framework. Leadersh Q. (2018) 29:89–104. doi: 10.1016/j.leaqua.2017.12.009, PMID: 29527338

[47] Vroom VH Jago AG. The role of the situation in leadership. Am Psychol. (2007) 62:17–24. doi: 10.1037/0003-066X.62.1.17, PMID: 17209676

[48] Andenoro A Wasserman J Newsome WJ. Building moral imagination, emotionally engaged thinking, and adaptive leadership capacity in leadership learners through the power of the holocaust. J Charact Leadersh Dev. (2019) 6:134–50.

[49] Northouse PG. Leadership: theory and practice. 8th ed. Thousand Oaks, CA: SAGE (2019). 1678196200 p.

[50] Oc B. Contextual leadership: a systematic review of how contextual factors shape leadership and its outcomes. Leadersh Q. (2018) 29:218–35. doi: 10.1016/j.leaqua.2017.12.004, PMID: 33383271

[51] Alessio F Finstad GL Giorgi G Lulli LG Traversini V Lecca LI. Intrapreneurial self-capital. An overview of an emergent construct in organizational behaviour. Calitatea. (2019) 20:156–62.

[52] Tucker S Chmiel N Turner N Hershcovis MS Stride CB. Perceived organizational support for safety and employee safety voice: the mediating role of coworker support for safety. J Occup Health Psychol. (2008) 13:319–30. doi: 10.1037/1076-8998.13.4.319, PMID: 18837627

[53] Afsar B Shahjehan A Cheema S Javed F. The effect of perceiving a calling on Pakistani nurses’ organizational commitment, organizational citizenship behavior, and job stress. J Transcult Nurs. (2018) 29:540–7. doi: 10.1177/1043659618761531, PMID: 29557278

[54] Ma Z Khan HS Chughtai MS Li M Ge B Qadri SU. A review of supervisor–subordinate Guanxi: current trends and future research. Sustainability. (2023) 15:795. doi: 10.3390/su15010795

[55] Khan HS Guangsheng Y Chughtai MS Cristofaro M. Effect of supervisor-subordinate Guanxi on employees work behavior: an empirical dynamic framework. J Innov Knowl. (2023) 8:100360. doi: 10.1016/j.jik.2023.100360

[56] Jones RA Jimmieson NL Griffiths A. The impact of organizational culture and reshaping capabilities on change implementation success: the mediating role of readiness for change. J Manag Stud. (2005) 42:361–86. doi: 10.1111/j.1467-6486.2005.00500.x

[57] Holt DT Armenakis AA Feild HS Harris SG. Readiness for organizational change: the systematic development of a scale. J Appl Behav Sci. (2007) 43:232–55. doi: 10.1177/0021886306295295, PMID: 32368619

[58] Holt DT Bartczak SE Clark SW Trent MR. The development of an instrument to measure readiness for knowledge management. Knowl Manag Res Pract. (2007) 5:75–92. doi: 10.1057/palgrave.kmrp.8500132

[59] Luo C-Y Tsai C-HK Su C-HJ Kim HJ Gao J-L Chen M-H. How does hotel employees’ psychological capital promote adaptive performance? The role of change readiness. J Hosp Tour Manag. (2022) 51:491–501. doi: 10.1016/j.jhtm.2022.05.006

[60] Alolabi YA Ayupp K Dwaikat MA. Issues and implications of readiness to change. Adm Sci. (2021) 11:140. doi: 10.3390/admsci11040140, PMID: 37921103

[61] Endrejat PC Klonek FE Müller-Frommeyer LC Kauffeld S. Turning change resistance into readiness: how change agents’ communication shapes recipient reactions. Eur Manag J. (2021) 39:595–604. doi: 10.1016/j.emj.2020.11.004

[62] Rahi S Alghizzawi M Ahmad S Khan MM Ngah AH. Does employee readiness to change impact organization change implementation? Empirical evidence from emerging economy. Int J Ethics Syst. (2021) 38:235–53. doi: 10.1108/IJOES-06-2021-0137

[63] Mrayyan MT Nijmeh A-A Al-Rawashdeh S Algunmeeyn A Abunab HY Wafa’a WO. How does authentic leadership influence the safety climate in nursing? BMJ Leader. (2023) 7:189–95. doi: 10.1136/leader-2022-000677, PMID: 37192096 PMC12038108

[64] Arthur YA Boardman GH Morgan AJ McCann TV. Effectiveness of a problem-solving, story-bridge mental health literacy programme in improving Ghanaian community leaders’ attitudes towards people with mental illness: a cluster randomised controlled trial. Issues Ment Health Nurs. (2020) 42:332–45. doi: 10.1080/01612840.2020.1799273, PMID: 32877258

[65] Zadow AJ Dollard MF Mclinton SS Lawrence P Tuckey MR. Psychosocial safety climate, emotional exhaustion, and work injuries in healthcare workplaces. Stress Health. (2017) 33:558–69. doi: 10.1002/smi.2740, PMID: 28127855

[66] Neal A Griffin MA. A study of the lagged relationships among safety climate, safety motivation, safety behavior, and accidents at the individual and group levels. J Appl Psychol. (2006) 91:946–53. doi: 10.1037/0021-9010.91.4.946, PMID: 16834517

[67] Hall GB Dollard MF Coward J. Psychosocial safety climate: development of the PSC-12. Int J Stress Manag. (2010) 17:353–83. doi: 10.1037/a0021320, PMID: 31905608

[68] Stouten J Rousseau DM De Cremer D. Successful organizational change: integrating the management practice and scholarly literatures. Acad Manag Ann. (2018) 12:752–88. doi: 10.5465/annals.2016.0095

[69] Acton KS. School leaders as change agents: do principals have the tools they need? Manag Educ. (2021) 35:43–51. doi: 10.1177/0892020620927415

[70] Gilley A Thompson J Gilley JW. Leaders and change: attend to the uniqueness of individuals. J Appl Manag Entrep. (2012) 17:69–83.

[71] Seibert SE Kraimer ML Crant JM. What do proactive people do? A longitudinal model linking proactive personality and career success. Pers Psychol. (2001) 54:845–74. doi: 10.1111/j.1744-6570.2001.tb00234.x

[72] Zampetakis LA. The role of creativity and proactivity on perceived entrepreneurial desirability. Think Skills Creat. (2008) 3:154–62. doi: 10.1016/j.tsc.2008.07.002

[73] Xuesheng D Xintao Z. An empirical investigation of the influence of safety climate on safety citizenship behavior in coal mine. Procedia Eng. (2011) 26:2173–80. doi: 10.1016/j.proeng.2011.11.2422

[74] Jiang W Gu Q. A moderated mediation examination of proactive personality on employee creativity: a person-environment fit perspective. J Organ Chang Manag. (2015) 28:393–410. doi: 10.1108/JOCM-05-2014-0088

[75] Kisamore JL Liguori EW Muldoon EW Jeffrey Jawahar IM. Keeping the peace: an investigation of the interaction between personality, conflict, and competence on organizational citizenship behaviors. Career Dev Int. (2014) 19:244–59. doi: 10.1108/CDI-09-2013-0115

[76] Grant AM Ashford SJ. The dynamics of proactivity at work. Res Organ Behav. (2008) 28:3–34. doi: 10.1016/j.riob.2008.04.002, PMID: 38169894

[77] Ahmad I Gao Y Su F Khan MK. Linking ethical leadership to followers’ innovative work behavior in Pakistan: the vital roles of psychological safety and proactive personality. Eur J Innov Manag. (2023) 26:755–72. doi: 10.1108/EJIM-11-2020-0464

[78] Philip J. A multi-study approach to examine the interplay of proactive personality and political skill in job crafting. J Manag Organ. (2023) 29:207–26. doi: 10.1017/jmo.2021.1

[79] Shi Y Zhou J-x Shi J-l Pan J-F Dai J-y Gao Q. Association between proactive personality and professional identity of nursing undergraduates: the mediating role of resilience and irrational belief. Nurse Educ Pract. (2023) 71:103729. doi: 10.1016/j.nepr.2023.103729, PMID: 37506426

[80] Doğanülkü HA Korkmaz O. The role of proactive personality and general self-efficacy in proactive career behavior: a mediation model. Int J Educ Vocat Guid. (2023):1–25. doi: 10.1007/s10775-023-09597-9 PMC10208558 37360274

[81] Wang J Kim T-Y Tekleab A Gilbreath B. The interplay between perceived support and proactive personality: effects on self-verification perceptions and emotions. Int J Hum Resour Manag. (2023) 34:2832–55. doi: 10.1080/09585192.2022.2095223

[82] Podsakoff PM MacKenzie SB Podsakoff NP. Sources of method bias in social science research and recommendations on how to control it. Annu Rev Psychol. (2012) 63:539–69. doi: 10.1146/annurev-psych-120710-100452, PMID: 21838546

[83] Podsakoff PM MacKenzie SB Lee J-Y Podsakoff NP. Common method biases in behavioral research: a critical review of the literature and recommended remedies. J Appl Psychol. (2003) 88:879–903. doi: 10.1037/0021-9010.88.5.879, PMID: 14516251

[84] Rosenthal R Rosnow RL. Essentials of behavioral research: methods and data analysis. 3rd ed. Boston: McGraw-Hill (2008). 1698941890 p.

[85] Ullah Z Sulaiman MABA Ali SB Ahmad N Scholz M Han H. The effect of work safety on organizational social sustainability improvement in the healthcare sector: the case of a public sector hospital in Pakistan. Int J Environ Res Public Health. (2021) 18:6672. doi: 10.3390/ijerph18126672, PMID: 34205758 PMC8296406

[86] Vaishnavi V Suresh M Dutta P. A study on the influence of factors associated with organizational readiness for change in healthcare organizations using TISM. Benchmarking. (2019) 26:1290–313. doi: 10.1108/BIJ-06-2018-0161

[87] Armstrong JS Overton TS. Estimating nonresponse bias in mail surveys. J Mark Res. (1977) 14:396–402. doi: 10.1177/002224377701400320, PMID: 38032037

[88] Harman D. A single factor test of common method variance. J Psychol. (1967) 35:359–78.

[89] Chughtai MS Syed F Naseer S Chinchilla N. Role of adaptive leadership in learning organizations to boost organizational innovations with change self-efcacy. Curr Psychol. (2023):1–20. doi: 10.1007/s12144-023-04669-z PMC10132955 37359696

[90] Chughtai MS Syed F Naseer S. Learning organizations and employees’ outcomes: a perspective of psychosocial safety climate. NICE Res J. (2022) 15:15–46. doi: 10.51239/nrjss.vi.329

[91] Chughtai MS HSUD Khan Zhiqiang M Mingxing L. Surmounting muddled pathways: adaptive leadership and psychosocial safety climate approach. Academy of Management Proceedings. (2022) Academy of Management Briarcliff Manor, Briarcliff Manor, NY; 17313.

[92] Chrisanty FN Gunawan MS Wijayanti RW Soetjipto BW. The role of transformational entrepreneurship, readiness to change and counterproductive work behavior in enhancing employee performance. Organ. (2021) 54:63–81. doi: 10.2478/orga-2021-0005

[93] Olafsen AH Nilsen ER Smedsrud S Kamaric D. Sustainable development through commitment to organizational change: the implications of organizational culture and individual readiness for change. J Work Learn. (2021) 33:180–96. doi: 10.1108/JWL-05-2020-0093

[94] Shin I Kim M. Proactive personality as a critical condition for seeking advice and crafting tasks in ambiguous roles. Behav Sci. (2022) 12:481. doi: 10.3390/bs12120481, PMID: 36546964 PMC9774385

[95] Meng X Chan AH. Cross-regional research in demographic impact on safety consciousness and safety citizenship behavior of construction workers: a comparative study between mainland China and Hong Kong. Int J Environ Res Public Health. (2022) 19:12799. doi: 10.3390/ijerph191912799, PMID: 36232095 PMC9566649

[96] Zhai H Li M Hao S Chen M Kong L. How does metro maintenance staff’s risk perception influence safety citizenship behavior—the mediating role of safety attitude. Int J Environ Res Public Health. (2021) 18:5466. doi: 10.3390/ijerph18105466, PMID: 34065328 PMC8160694

[97] Vakola M. What’s in there for me? Individual readiness to change and the perceived impact of organizational change. Leadersh Organ Dev J. (2014) 35:195–209. doi: 10.1108/LODJ-05-2012-0064

[98] Li W-D Fay D Frese M Harms PD Gao XY. Reciprocal relationship between proactive personality and work characteristics: a latent change score approach. J Appl Psychol. (2014) 99:948–65. doi: 10.1037/a0036169, PMID: 24635530

[99] Hofmann DA Morgeson FP Gerras SJ. Climate as a moderator of the relationship between leader-member exchange and content specific citizenship: safety climate as an exemplar. J Appl Psychol. (2003) 88:170–8. doi: 10.1037/0021-9010.88.1.170, PMID: 12675404

[100] Geier MT. Leadership in extreme contexts: transformational leadership, performance beyond expectations? J Leadersh Organ Stud. (2016) 23:234–47. doi: 10.1177/1548051815627359

[101] Valle M Kacmar M Andrews M. Ethical leadership, frustration, and humor: a moderated-mediation model. Leadersh Organ Dev J. (2018) 39:665–78. doi: 10.1108/LODJ-02-2018-0083

[102] Lt H Bentler PM. Cutoff criteria for fit indexes in covariance structure analysis: conventional criteria versus new alternatives. Struct Equ Model Multidiscip J. (1999) 6:1–55. doi: 10.1080/10705519909540118

[103] Tanaka JS. Multifaceted conceptions of fit in structural equation models In: Bollen KA Long JS, editors. Testing structural equation models. Newbury Park, CA: SAGE (1993). 10–39.

[104] Hair JF Black WC Babin BJ Anderson RE. Multivariate data analysis. 7th ed. Upper Saddle River, NJ: Prentice Hall (2010). 1678196176 p.

[105] Hair JF Risher JJ Sarstedt M Ringle CM. When to use and how to report the results of PLS-SEM. Eur Bus Rev. (2019) 31:2–24. doi: 10.1108/EBR-11-2018-0203, PMID: 38163164

[106] Sekaran U Bougie R. Research methods for business: a skill-building approach. 5th ed. United Kingdom: John Wiley & Sons, Ltd (2010). 1595601313 p.

[107] Ratner B. The correlation coefficient: its values range between +1/−1, or do they? J Target Meas Anal Mark. (2009) 17:139–42. doi: 10.1057/jt.2009.5, PMID: 38179581

[108] Hayes AF. Introduction to mediation, moderation, and conditional process analysis. 2nd ed. New York: The Guilford Press (2018). 1595601312 p.

[109] Hernández-Santiago N Pérez-Rivera M. Adaptive leadership as a method to overcome organizational crisis: a Puerto Rican study. Forum Empresarial. (2022) 26:99–123. doi: 10.33801/fe.v26i2.19883

[110] Nelson T Squires V. Addressing complex challenges through adaptive leadership: a promising approach to collaborative problem solving. J Leaders Educ. (2017) 16:111–23. doi: 10.12806/V16/I4/T2

[111] Radian NN Mangundjaya WL. Individual readiness for change as mediator between transformational leadership and commitment affective to change. Jurnal Manajemen Aset Infrastruktur Fasilitas. (2019) 3:1–12.

[112] Afsharian A Dollard M Miller E Puvimanasinghe T Esterman A De Anstiss H. Refugees at work: the preventative role of psychosocial safety climate against workplace harassment, discrimination and psychological distress. Int J Environ Res Public Health. (2021) 18:10696. doi: 10.3390/ijerph182010696, PMID: 34682442 PMC8535317

[113] Idris MA Abdullah SS. Psychosocial safety climate improves psychological detachment and relaxation during off-job recovery time to reduce emotional exhaustion: a multilevel shortitudinal study. Scand J Psychol. (2021) 63:19–31. doi: 10.1111/sjop.12789 34807489

[114] Abdul HR. The role of employees’ technology readiness, job meaningfulness and proactive personality in adaptive performance. Sustainability. (2022) 14:15696. doi: 10.3390/su142315696

[115] Asghar F Mahmood S Iqbal Khan K Gohar Qureshi M Fakhri M. Eminence of leader humility for follower creativity during COVID-19: the role of self-efficacy and proactive personality. Front Psychol. (2022) 12:1–11. doi: 10.3389/fpsyg.2021.790517 PMC8776629 35069376

